# 4D imaging reveals stage dependent random and directed cell motion during somite morphogenesis

**DOI:** 10.1038/s41598-018-31014-3

**Published:** 2018-08-23

**Authors:** James McColl, Gi Fay Mok, Anna H. Lippert, Aleks Ponjavic, Leila Muresan, Andrea Münsterberg

**Affiliations:** 1 School of Biological Sciences, Cell and Developmental Biology, University of East Anglia, Norwich Research Park, Norwich, NR4 7TJ UK; 2 Chemistry Department, University of Cambridge, Lensfield Road, Cambridge, CB2 1EW UK; 3 Cambridge Advanced Imaging Centre (CAIC), Downing Street, Cambridge, CB2 3DY UK

## Abstract

Somites are paired embryonic segments that form in a regular sequence from unsegmented mesoderm during vertebrate development. Although transient structures they are of fundamental importance as they generate cell lineages of the musculoskeletal system in the trunk such as cartilage, tendon, bone, endothelial cells and skeletal muscle. Surprisingly, very little is known about cellular dynamics underlying the morphological transitions during somite differentiation. Here, we address this by examining cellular rearrangements and morphogenesis in differentiating somites using live multi-photon imaging of transgenic chick embryos, where all cells express a membrane-bound GFP. We specifically focussed on the dynamic cellular changes in two principle regions within the somite, the medial and lateral domains, to investigate extensive morphological transformations. Furthermore, by using quantitative analysis and cell tracking, we capture for the first time a directed movement of dermomyotomal progenitor cells towards the rostro-medial domain of the dermomyotome, where skeletal muscle formation initiates.

## Introduction

Embryonic morphogenesis involves dramatic tissue deformation and growth, which often occurs rapidly over short time-scales. It is implicit that tissue deformations are driven by local cellular activities, including cell proliferation, changes in morphology and/or size, and cell rearrangements. However, it has been challenging to image, capture and quantify these processes in live tissues.

Somites are transient, epithelial, near spherical structures that form during vertebrate development from the presomitic mesoderm (PSM) in a regular sequence and with a rostro-caudal progression^1^. Somites can be staged based on morphological landmarks and age of development, using roman numerals^2^. Newly formed somites consist of a ball of epithelial cells surrounding a central cavity, the somitocoel, which is filled with mesenchymal cells (stages I–III). As they differentiate, these paired body segments dissociate ventrally (from stage IV) and epithelial-to-mesenchymal transition (EMT) leads to formation of the sclerotome, the source of the axial skeleton. The dorsal somite remains epithelial and produces the dermomyotome and myotome, the source of all trunk and limb skeletal muscles^2,3^. Signalling and genetic control of cell lineage specification is well characterised^4–6^. For example, expression of the first myogenic marker, the transcription factor Myf5, is first detectable in the medial wall of epithelial somites^7^. However, surprisingly very little is known about how individual cell dynamics and cellular rearrangements drive morphogenesis within the somite during its differentiation, for example during the emergence of the myotome. An improved and greater understanding of these processes may also benefit the derivation of musculoskeletal lineages from pluripotent stem cells^8^.

Along the rostro-caudal axis, each individual somite is flanked by neighbouring somites; other adjacent tissues on the medial, lateral, dorsal and ventral sides are the neural tube (future spinal cord), the intermediate and lateral plate mesoderm, the surface ectoderm and the endoderm respectively. Signalling molecules derived from many of these tissues govern the specification of somite cells towards particular fates^9–20^. In addition, these flanking tissues impose rigidity and mechanical constraints, which are likely to contribute to somite morphogenesis, however, this remains to be tested. Whilst examination of fixed tissues has contributed to our current understanding of somite morphology during somite differentiation, the cellular dynamics driving somite morphogenesis have not been investigated in real time.

The medial domain of the somite, closest to and running parallel to the neural tube, is particularly important for the formation of skeletal muscle. It is here that, the early, epaxial myotome first forms. Cells delaminate from the medial lip of the epithelial dermomyotome (the DML) and navigate, as myoblasts, ventral to the dermomyotome where they differentiate. Subsequently cells enter from all dermomyotomal lips, at later stages of somite differentiation. The timing of this process has been extensively characterized using intricate cell labelling, for example using focal Dye injections or GFP electroporations^21–25^, and is reviewed in^26^.

Cell proliferation within the dermomyotome, including in its lips, contributes to its growth^23,27,28^. In epithelial somites, most cells were labelled following a short pulse of BrdU, with exception of some cells located in the medial wall of the somite abutting the neural tube^29^, suggesting they may be post-mitotic or exhibit a slower rate of cell proliferation. Tracing of DiI labelled cells from the medial domain of epithelial somites to subsequent locations, detected 6, 12 and 18 hours after labelling, indicated that these cells migrate into the rostral somite half and then elongate towards its caudal border^30^. This observation suggests that medial wall derived cells make a discrete contribution to the early myotome that is distinct from the DML^26^. Myoblasts extending caudally from the rostral somite edge have also been observed using desmin staining^30,31^. The expression of skeletal muscle specific markers also identifies the location of early myoblasts^7,32^. However, cellular rearrangements during the emergence of the myotome have not been observed in real time. Furthermore, the potential contribution of localized cell proliferation to somite morphogenesis has not yet been evaluated.

In addition, the role of force and cell flow is important for somite development. Discreet patches of laminin within the extracellular matrix (ECM) for example have been shown to form during early somite development. As the matrix develops it provides activating and constraining force regions^33,34^. Cell movement and cell expansion will generate forces which will push, pull and direct the position of cells and therefore shape tissues. This creates tension and stress responses from cells and surrounding matrices, and with surface tension thought to play a small role in development^35^ direct force transfer between cells is most likely a primary mechanism.

This work sought to characterize and quantitate the behaviour of individual cells in real time, in a living, developing somite. Thus far our insights regarding the dynamic cell re-arrangements during somite development are limited as almost everything has been derived from the analysis of fixed samples. Using multi-photon time-lapse microscopy and quantitative analyses, we measure somite growth and establish that it is mainly driven by cell proliferation. Interestingly, proliferation is not uniform and instead asymmetrically distributed across the somite stages examined. We focus on early stages, where the somite is still epithelial, and on intermediate (mid) stages, where it has dissociated ventrally and where the dorsomedial lip (DML) of the epaxial dermomyotome begins to form. We show that at these early and intermediate stages medial somite regions contain fewer cells. We also identify a population of larger cells in this region. Using correlation analyses we show in living tissue that morphogenetic changes are most pronounced in intermediate stage somites. These changes correlate with an increase in the number of proliferating cells. Our in-depth analysis also reveals that dermomyotome cells undergo a collective, directed motion towards the emerging epaxial dermomyotome lip, where myogenic cells are known to enter the myotome. Taken together, we identify discrete cell populations and cellular drivers of early somite morphogenesis and propose a model whereby the nascent dermomyotome lip creates a sink towards which the cells move due to local cell proliferation and constraints imposed by surrounding tissues.

## Results

### Somite growth rate

**Figure 1:**
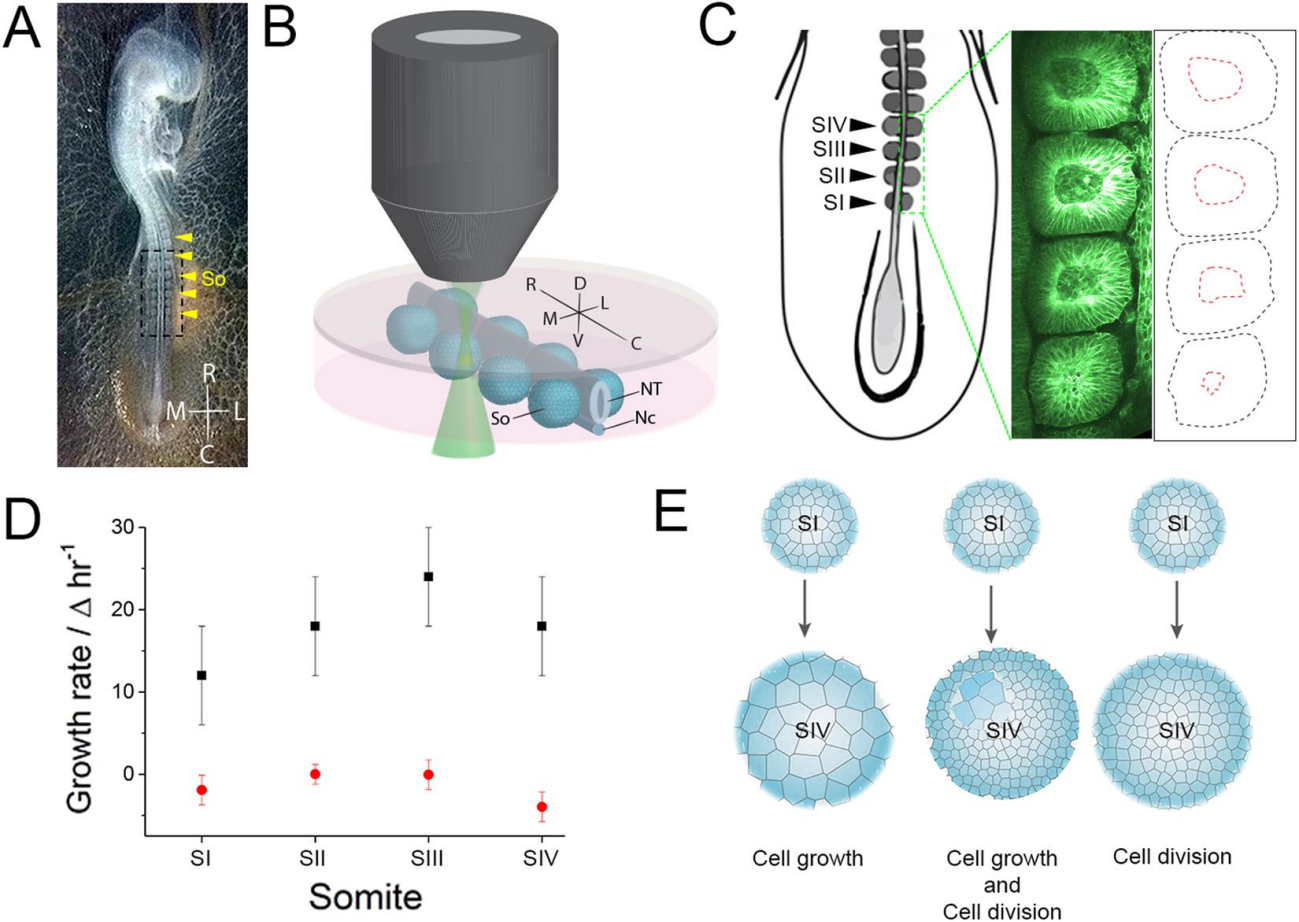
Imaging and measuring somite growth. Somites from 2 day-old memGFP transgenic chicken embryos were dissected as indicated by black dotted line box (**A**). Somites were dissected, positioned and set into an agarose:media gel in chambers and imaged by 2-photon microscopy (**B**). The volume of the four newest formed somites (SI, SII, SII and SIV), both the whole somite (black dotted lines) and the somitocoel (red dotted lines), were measured and calculated (**C,D**, n = 4). The growth rates for individual somites were calculated and plotted with an average growth rate of 19 +/− 4 µm^3^ hr^−1^ determined for the somite (**D**). Growth can be modelled as either individual cell growth, cell division or a combination of both (**E**). So, somites; NT, neural tube; Nc, notochord; R, rostral; C, caudal; M, medial; L, lateral; D, dorsal; V, ventral. Errors are SD.

To determine the extent to which migration, proliferation and cell growth contribute to somite growth and changes in tissue shape, we sought to image somite growth directly. Using multi-photon microscopy, we determined the spatiotemporal distribution of cells and their behaviour. Multiple somites were excised intact and oriented together with intact flanking tissues in humidified imaging chambers (Fig. 1A,B). We imaged the four newest somites formed in the caudal region of HH stage 14/15 embryos^36^ and examined their change in overall volume, and separately measured the change in size of the central cavity (somitocoel) (Fig. 1C,D). Firstly, the growth rate of individual somites was determined over 180 minutes, which is equivalent to approximately two somite stages. All somites increased in volume with a rate of 19 +/− 4 µm^3^ hr^−1^ (Fig. 1D). In contrast, the size of the somitocoel remained constant during this period (126 +/− 4 µm^3^ at time 0 and 124 +/− 4 µm^3^ at 180 minutes). Based on this data, we modelled somite growth as a collection of spherical structures to determine the number of cell divisions required if growth is solely dependent on proliferation (Fig. 1E). For a growth increase of 19 µm^3^ hr^−1^ the division rate of somite cells would need to be 3 new cells hr^−1^ for an average cell size of 6 µm^37^. Alternatively, the volume change may be due to increase in cell size, or a combination of both proliferation and size increase (Fig. 1E).

### Characterization of cellular features in medial and lateral somite domains

**Figure 2:**
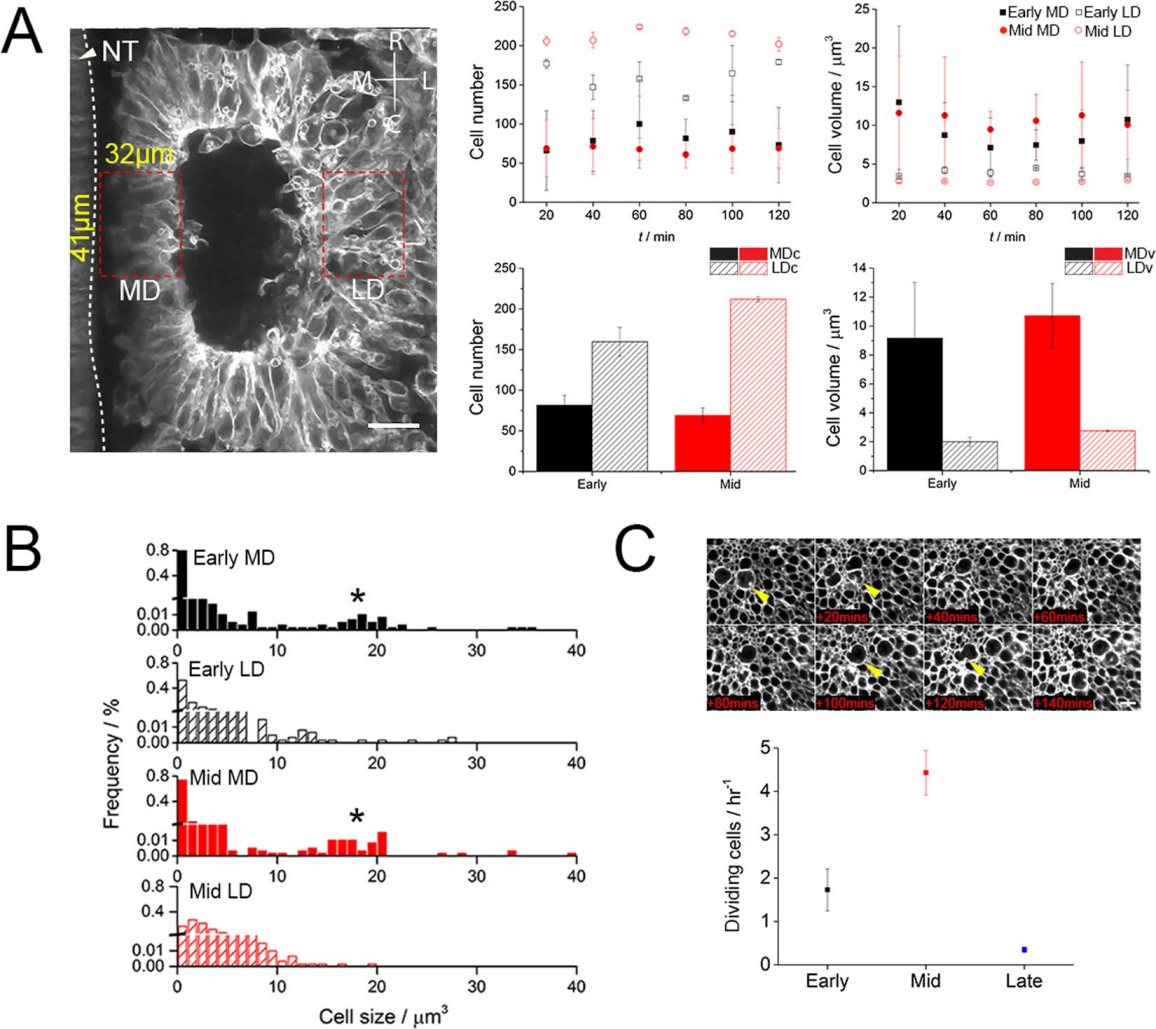
Cell size and number are heterogeneous across the somite. Cell number and volume were determined over time for early (black square) and mid (red circle) stage somites at the medial domain (MD) and lateral domain (LD). Measurements for the domains: width 32 µm, length 41 µm, depth 20 µm. Average comparisons for all data sets, MDc and LDc denotes cell number in MD and LD; MDv and LDv denote cell volume in MD and LD (**A**). Cell volume histograms for MD (solid fill) and LD (pattern fill) for early (black) and mid-stage (red) somites (**B**) with asterix indicating larger cells. Cell proliferation rate was determined by counting dividing cells (yellow arrow head). Cell proliferation at the LD and proliferation rates for early (black, n = 3), mid (red, n = 4) and late (blue, n = 3) stages were measured. NT, neural tube; R, rostral; C, caudal; M, medial; L, lateral; D, dorsal; V, ventral. Errors are SD and Scale bar 20 µm.

We next investigated proliferation behaviour and cell size to determine their potential contributions to overall somite growth. We focussed on defined regions, the medial (MD) and lateral (LD) somite domains that are flanking the somitocoel of early, still epithelial somites and measured a range of parameters including the number and size of cells over time (120 min) (Fig. 2A). The same parameters were measured in equivalent medial and lateral domains of mid-stage somites that have dissociated ventrally and where the dermomyotome lips are forming. The difference between early and mid-stage somites is approximately four to five somite stages. The number of cells in the medial domain is similar for both early and mid-stages, 81 +/− 12 and 68 +/− 3, respectively. In contrast, in both early and mid-stage somites the number of cells in the lateral domain is higher. Furthermore, a higher number of cells was found in the LD of mid-stage somites, 212 +/− 8, when compared to the LD of early stage somites, 160 +/− 18 (Fig. 2A). When examining cell size, we found a more uniformly sized population of smaller cells at the LD compared to the MD where cell size was more variable in both early and mid-stage somites (Fig. 2A). Quantification of cell size distribution revealed that the medial domain comprises two cell populations with a discrete size difference (Fig. 2B). In addition, we assessed the number of cell division events by counting large rounded cells throughout somites at early, mid and also at later stages of development, where the myotome had begun to form (Fig. 2C). We find that the cell division rate was much greater in mid stage somites than in early or late stage somites (p value = 4.7 e-8 for mid against early, and p value = 1.3 e-4 for mid against late, two tailed t-test). The cell division rates in early and late somites were not significantly different (p value = 0.2) (Fig. 2C).

### The rate of morphological change is greater at the mid somite stage

**Figure 3:**
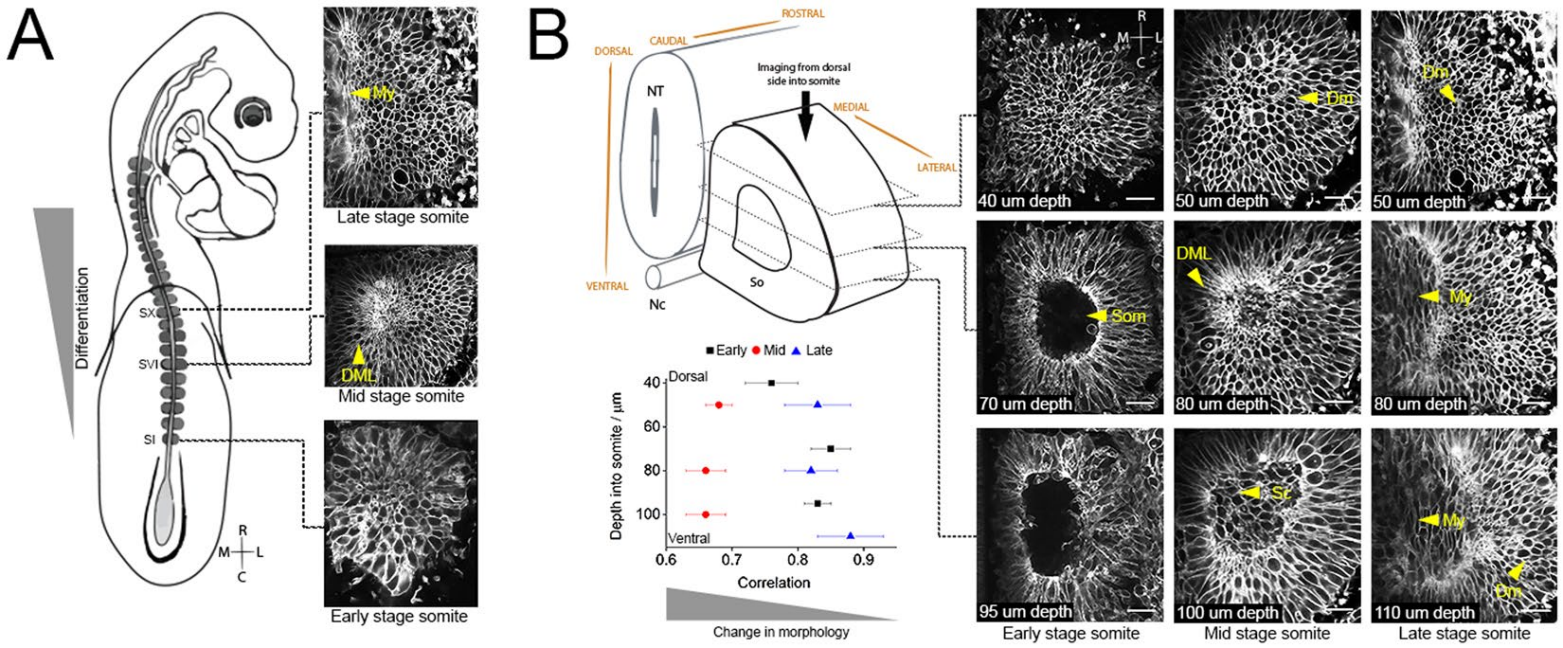
Greatest morphological changes are at the mid stage somite. Early (SI), mid (SVI) and late (SX) stage somites were individually imaged and analysed (**A**). Top, middle and bottom of early (black square, n = 3), mid (red circle, n = 4) and late (blue triangle, n = 3) stage somites were correlated by overlaying successive images between t0 and t180 min (**B**). A higher correlation value indicates less change over time. My, myotome; DML, dorso-medial lip; Dm, dermomyotome; Som, somitocoel; Sc, sclerotome. Scale bars 10 µm.

Next, we examined whether the cellular parameters measured correlate with morphological changes of somites as they mature. Somite morphology is indicative of its developmental stage and position along the embryonic axis, with more recently formed somites less differentiated (early stage) compared to more rostral somites (late stage) (Fig. 3A and Movies V1–3. We investigated if the rate of change in somite morphology was different for somites as they progress in their differentiation. To do this we compared somites from early, mid and late stages along the embryonic axis. As before, the early stage somites (SI–SIII) were epithelial and the mid stage somites had dissociated ventrally and the DML was forming (SIV–SVI). The late stage somites imaged had a myotome layer (SIX–SXI). For each stage we determined the change in somite morphology over 180 minutes (Fig. 3B). The distribution of morphology changes were assessed through the tissue by comparing slices from the dorsal, centre and ventral sections of individual somites from early, mid and late stages. By calculating the correlation coefficient for each slice of each somite we are able to calculate how much the somite changes, with a higher correlation coefficient meaning less change. Interestingly, we found that morphology changes were not linear and mid stage somites changed most, in all regions of the somite: dorsal, centre and ventral. By contrast, the early and late stage somites changed less with greater change observed in the dorsal region of early somites (Fig. 3B). Thus, mid stage somites were more dissimilar throughout the whole somite, indicating a greater rate of change during the time window measured. We include Movies (V4 and V5) from 80 and 100 µm depth of mid stage somites.

### In mid stage somites dermomyotome cells display a concerted movement towards rostro-medial regions

**Figure 4:**
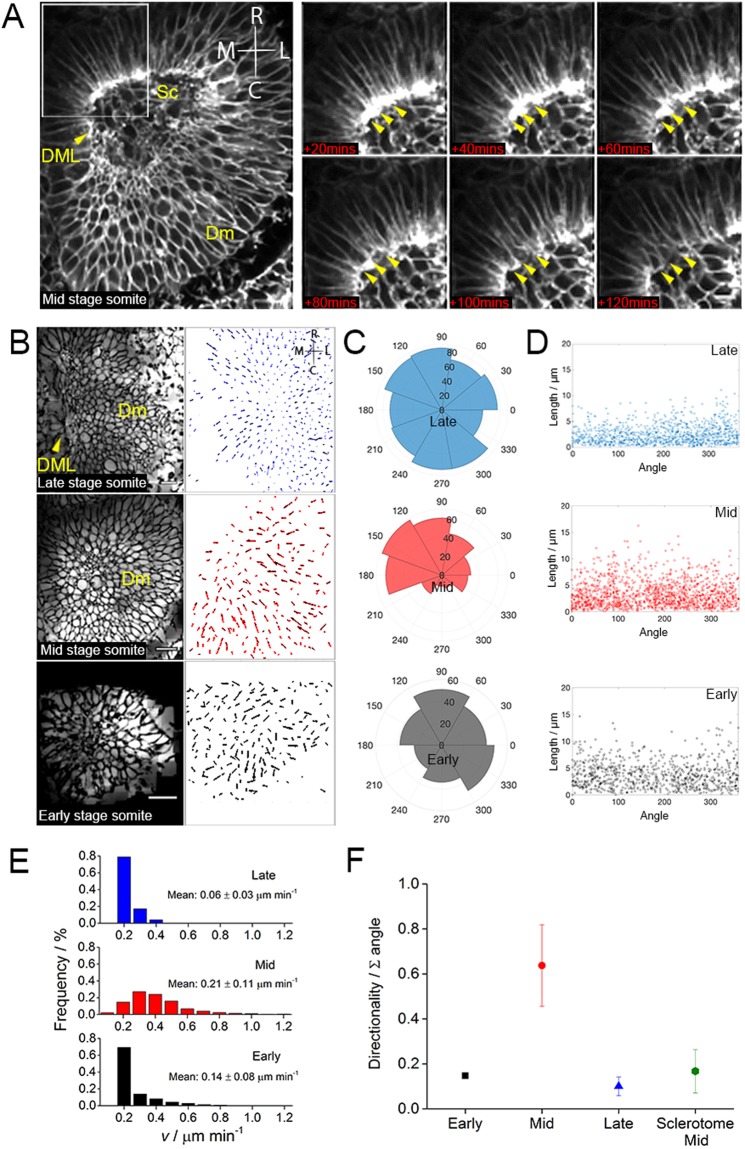
Directional concerted cell movement in mid-stage somites towards the rostro-medial region. Observation of dissociating cells (yellow arrowheads in rostro-medial region of somite. Scale bar is 10 µm (**A**). Inverted images of dermomyotome are used to track individual cells in early, mid and late stage somites. Scale bars 20 µm (**B**). Angle distribution for individual tracks of early (black, n = 3), mid (red, n = 4) and late (blue, n = 3) stage somites (**C**). Length versus angle plots for all tracks (**D**). Track speed for early (black), mid (red) and late (blue) stage somites measured (**E)**. Summing all tracks gives directionality for early (black), mid (red) and late (blue) stages somites of cells in dermomyotome and cells in mid-stage somite sclerotome (**F**). Directional concerted cell movement model for mid stage somites (**G**). DML, dorso-medial lip; Sc, sclerotome; Dm, dermomyotome.

To further understand the mechanisms underlying the greater rate of change in mid-stage somites, we characterized cell behaviours in more detail. In the rostro-medial corner of mid stage somites we found detaching cells, which suggests an epithelial to mesenchymal transition in this region (Fig. 4A). To determine the cellular processes that may contribute and potentially drive this behaviour we tracked individual cell movement in the dermomyotome region in mid and late stage somites. We did the same in the equivalent dorsal region of early stage somites (i.e. above the somitocoel) (Fig. 4B and Movies V6–8). Using ImageJ and custom written Matlab software, we determined both the direction (Fig. 4C,D) and speed (Fig. 4E) of cells. In early-stage somites, movement of cells appeared random, with a mean velocity of 0.14 ± 0.08 µm min^−1^ (Fig. 4C–E). Interestingly, for mid-stage somites, we observed a concerted and directional movement of dermomyotome cells towards the rostro-medial quadrant (Fig. 4C,D), where the detaching cells are seen (Fig. 4A). By plotting the angle of the tracks as a function of their length, we find that the longest tracks are both directed and centred around the mean tracks angle for mid-stage somites (Fig. 4D). We also note that more cells were moving faster in mid-stage somites leading to an increased mean speed of 0.21 ± 0.11 µm min^−1^ (Fig. 4E). Plotting the mean direction of the tracks gives the overall directionality of dorsal or dermomyotome cells, respectively (Fig. 4F), which was higher for mid-stage somites compared to early and late stages. We asked whether the directional movements seen in mid stage somites were unique to dermomyotome cells. Measuring cells in the sclerotome showed that they exhibit low directionality suggesting random movements (Fig. 4F). In late-stage somites we observed cells moving inwards, towards the centre of the maturing somite, at a slower speed of 0.06 ± 0.03 µm min^−1^ (Fig. 4C–E). Due to the size of late stage somites it was not possible to measure sclerotome cells. Overall this correlates the greater rate of change in mid stage somites with a directed concerted movement of dermomyotome cells towards the rostro-medial region, where the cells are also dissociating.

## Discussion

**Figure 5:**
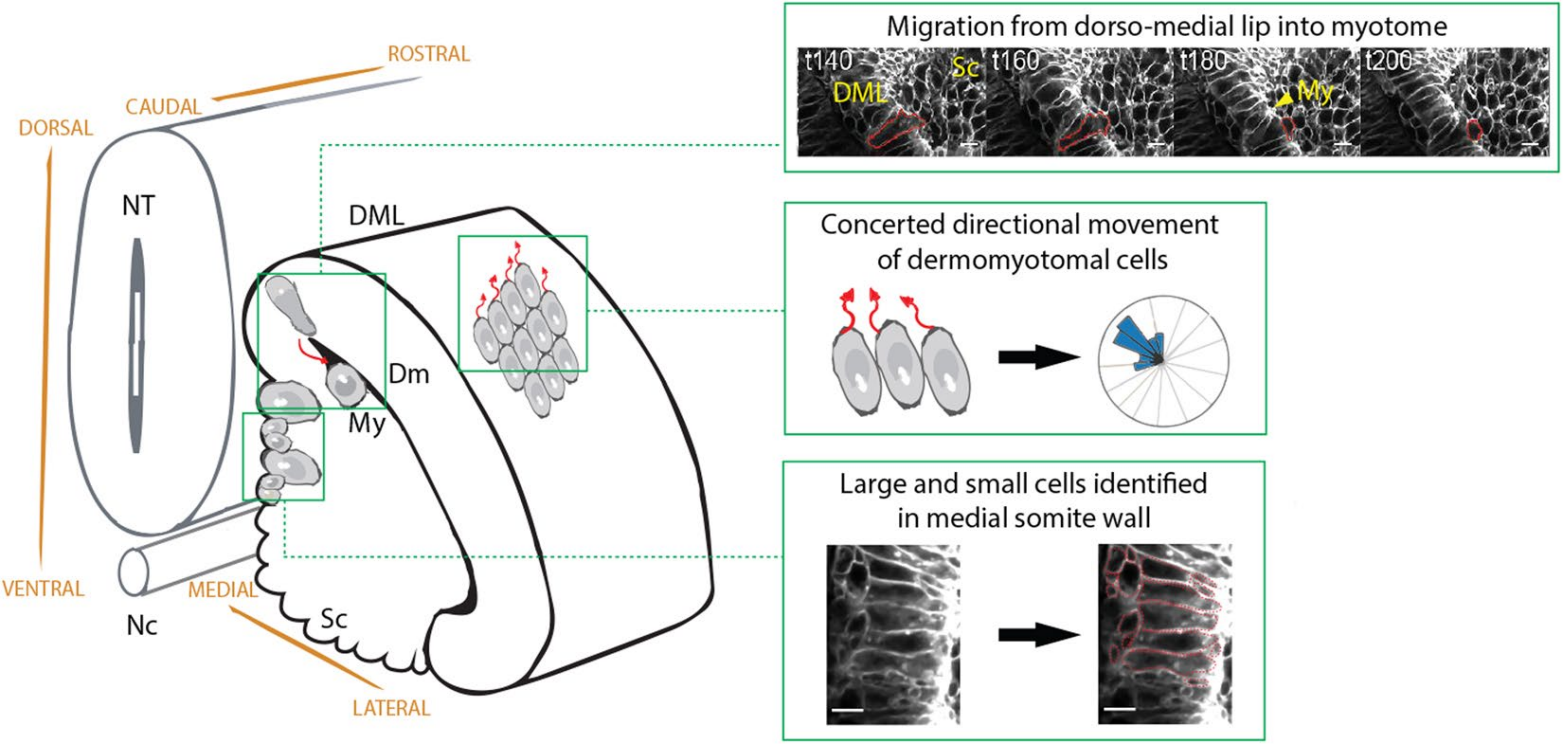
Summary of cellular dynamics in somites. Observation of epithelial cell moving from dorso-medial lip of dermomyotome into myotome. Large and small cells observed within the medial domain of somite. Directional concerted cell movement model for mid stage somites towards rostral-medial domain. DML, dorso-medial lip; Dm, dermomyotome; NT, neural tube; Nc, notochord; Sc, sclerotome; My, myotome. Scale bars 10 µm.

Somite differentiation is a dynamic process during the elongation of the vertebrate embryonic axis, involving rapid development in size and shape. Here we used real-time multi-photon imaging combined with precise quantitative analysis of cell size and number, in addition to detailed observations of localized cell behaviours to characterize this important process. This has provided novel insights into cellular mechanisms underlying somite morphogenesis. Our measurements reveal an unequal distribution of cell size and numbers, with a population of larger cells identified in the medial wall of early and mid-stage somites (Fig. 5). In addition, we uncover for the first time a directional movement of dermomyotome cells towards the rostro-medial region of the mid stage somite (Fig. 5), as well as suggesting a slower motion towards the centre of the dermomyotome at a later stage.

We used an *ex vivo* system with multiple somites surrounded by other native tissues, which are known to provide extrinsic signals and inductive cues for cell lineage determination^11,13,15,16,20,38–40^. The tissues maintained their structural integrity with active, viable cells that displayed coordinated behaviours and extended filopodia towards the surface ectoderm (not shown) as observed before^41^. We saw dividing cells and there was no indication of apoptosis. Furthermore, we observed in real time the morphological changes anticipated based on what is known from fixed whole embryo samples^25,29–31^. In mid stage somites cells delaminate in the rostro-medial region (Fig. 4A), and late stage somites formed a myotome layer (Fig. 3A,B and Movie V1–5). We also observed cells transitioning from the DML into the myotome (not shown) in agreement with previous reports (Fig. 5)^20,25^. Although overall tissue development appears to be somewhat slower, we believe that our quantitative *ex vivo* measurements closely reflect the behaviour in intact embryos.

Somites grow in size as they develop over a short period of time (Fig. 1D) and this growth could be due to an increase in cell size, cell proliferation or a combination of both (Fig. 1E). We determined somite growth rate of 19 µm^3^ min^−1^ for early somites, which equates to an increase of approximately 3 cells hr^−1^ if the growth is due to proliferation alone. The contribution of proliferation for early somites is 1.7 cells hr^−1^ (Fig. 2C), suggesting that at this stage approximately half of somite growth is achieved through proliferation and half through growth of individual cells (Fig. 1E).

We found that the number of cells increased with time. Interestingly, the greatest increase in the number of small sized cells was seen in lateral domains whereas greatest size increase occurred predominantly at the medial domain (MD) (Fig. 2A). This suggests a non-homogenous distribution of cell proliferation and is also in agreement with the presence of BrdU negative cells in the medial aspect of epithelial somites following pulse labelling^29^. We find that the MD comprises a discrete population of larger sized cells and it is tempting to speculate that these cells may be identical to the more slowly dividing, or potentially post-mitotic cells observed in the same location. However, we have not been able to observe these cells moving to the rostral somite half. The larger cells were seen at both early and mid-somite stages, and their number remains relatively constant (Fig. 2A). Proliferation rates increased for mid stage somites to 4.4 cells hr^−1^, and this is again likely to occur predominantly at the lateral domain (LD) of the somite, where the average number of cells increased to 212 (Fig. 2A). Together our data suggest that the majority of growth at the mid stage comes from proliferation.

Greater cell numbers and increased proliferation in the LD could also be responsible for the directed motion we observe of cells from lateral towards rostro-medial regions in mid-stage somites (Figs 4B–F, 5 and Movies V6–8). For late stage somites we found a low proliferation rate of 0.3 cells hr^−1^ and observed that these cells moved slowly, suggesting somite growth had almost stopped. It is likely that other processes such as maturation of the myotome and differentiation of muscle fibres begin to dominate at this stage (Figs 2C and 4C,E). It is also possible that myotome maturation may be reflected by the inward movement observed in the plane of the dermomyotome (4B).

We show that whole somite morphological changes were more pronounced in mid-stage somites and changes in cell number and size significantly contribute to this. Although early stage somites underwent less morphological change it is interesting that the dorsal region of the somite changed more and had a lower correlation coefficient (Fig. 3B). Based on the direction of tracked individual cells their motion appears random and we further observe that the longest tracks for this stage radiate across the somite in various directions (Fig. 4C,D). This behaviour could be explained if individual or small populations of cells move into different regions, as has been previously proposed^29,30^, however, we could not observe this directly. By contrast, for mid-stage somites we identified a directed movement of dermomyotomal cells towards the rostro-medial domain, where cells were detaching (Fig. 4A). The longest tracks were concentrated in a single direction (Fig. 4B–D). Desmin-positive myogenic cells are present in the rostro-medial, mid-stage somite^31^. In addition, DiI labelled cells that originate from the medial wall of early epithelial somites, have later been observed to generate myocytes extending from the rostral somite edge (after 18 hours)^30^. We have not been able to observe cells moving from the medial somite wall towards the rostral somite half, however, it is possible that single cell labelling combined with longer time-lapse imaging would be necessary to capture this. However, we do observe, in mid stage somites, a collective directed movement of dermomyotome cells from the caudal regions towards the rostro-medial domain (Fig. 4B–D), where cells delaminate (Fig. 4A) and the expression of myogenic markers is seen^7,30–32^. It is possible, that this movement may contribute to the fate decisions of these progenitors, for example by providing a mechanical stimulus as has been observed in stem cell differentiation^42,43^.

Furthermore, we propose a model whereby dermomyotomal progenitor cells may be moving towards a sink at the rostro–medial domain. Although outside the scope of this work, it is likely that multiple factors would be responsible for how these cells are able to orchestrate a precise motion towards a sink (Fig. 5). The larger cells in the MD, which may also undergo EMT and ingress, consistent with previous labelling studies^29,30^, may contribute to the sink and thus to the concerted cell movement identified here. Slightly later, medial lip cells have been shown to migrate via a so-called transition zone into the centre of the somite after receiving signals from the neural crest^20^. Finally, cells in the LD region may receive signals, including Wnt signals, from the overlying ectoderm^41,44,45^ to promote their proliferation, which in turn may create space constraints. This novel observation of a concerted motion of cells towards the rostro-medial domain of mid-stage somites, where cells are detaching, is exciting as it correlates with the presence of early myoblasts at the rostro-medial corner of the dissociating somite^7,32^. We speculate that this cellular motion, concomitant with EMT, may in fact contribute to the appearance of early myoblasts. In addition, we propose that asymmetrically distributed cell proliferation events, predominantly in the lateral region of the somite, are likely to play a key role by creating local forces and tension. We suggest that some of the cellular mechanisms revealed here, may be of more general importance for the shaping of tissues during embryonic development.

## Materials and Methods

### Embryo culture, dissection and mounting

Fertile eggs, transgenic for membrane-bound GFP (MemGFP, Roslin Institute, University of Edinburgh, UK) were incubated at 37 °C in a humidified incubator until HH14/15. Embryos were harvested in Glutamax Ham’s F12 media (cat. no. 31765) and explants containing somites, intermediate mesoderm, surface ectoderm, endoderm, notochord and neural tube were dissected using fine scalpels. We prepared low melting point agarose (Invitrogen cat. no. 16520-050, 2% w/v) in Glutamax Ham F12 media, then added 10% Fetal bovine serum and 1% penicillin/streptomycin (10,000 U/ml) after the agarose/media had cooled. The dissected tissue was positioned with the dorsal side towards the base of the glass slide, on non-coated 35 mm MatTek dishes with 7 mm glass diameter (P35G-0-7-C) and warm agarose/media added to the somite explants until fully covered. Dishes were placed on ice for 5 minutes before placing inside a humidified heat chamber (37 °C) for microscopy. The study was approved by the Animal Welfare & Ethical Review Board, School of Biological Sciences of the University of East Anglia and all procedures were performed in accordance with the relevant guidelines and regulations.

### Long-term two-photon microscopy

Individual somites were imaged as stacks of slices with each slice being 1000 × 1000 pixels (X = 250 nm Y = 250 nm Z = 510 nm). This enabled us to image an individual somite in 20 minute intervals, which included a 5-minute rest period. The number of slices was determined by the size of the somite at 20x magnification using a TriM Scope II (Labtec instruments) two-photon microscope. Initial laser power was increased exponentially through the stack to allow for signal loss due to deeper imaging. Low magnification images were recorded at 10x magnification.

### Tracking and image analysis

Images were first denoised using nd-safir, an image sequence denoising software to improve the signal to noise ratio of the images^46^. Following this, somites were aligned in Z manually and slices through the somite over time were selected using custom written Matlab code.

To determine the correlation coefficient images at time 0 and time 180 minutes were compared and correlated using Image_correlator plugin from ImageJ. Here, two images are compared pixel by pixel to determine their correlation. The value for each pixel is plotted as X for one image and Y for the other and once every pixel value is plotted the fit for the plots determines the correlation. If the two images are identical, the fit would be 1.

To track the cells, time slice stacks were inverted, bandpass filtered and background subtracted using a rolling ball radius of 20 pixels (ImageJ). Tracking was performed using the ImageJ trackmate plugin, simple LAP tracker^37^. We then use custom written matlab code to calculate the average direction for each track and it is these values which we use to plot the radial distribution and length versus angle plots. Summing all directionality values indicate how directed the cells were for early, mid and late stages. If the directions are random the directionality would be low as tracks moving in opposite directions would cancel out.

### Low mag whole somite size quantification

Somites were modelled as spheres over time by taking the central image of the somite and determining the perimeter using ImageJ. From the perimeter, we calculate the area of the somite as a sphere.

### 3D MD and LD quantification

Identical XY and Z regions from the medial domain (MD) and lateral domain (LD) were analysed on denoised somites. Early stage somites were imaged at a depth of 65–85 µm (10 µm either side half-way down the somite) and mid stage somites were imaged at a depth of 85–105 µm from the dorsal surface. The width measured was 32 µm and the rostro-caudal extent was 41 µm. The Real-time Accurate Cell-shape Extractor (RACE) analysis software was used to determine all cell parameters^47^ with custom written Matlab code used to extract and plot the data. To determine the cell parameters within the two fixed regions at the MD and LD of early and mid-stage somites we used the RACE algorithm and compared the two regions over time.

### Quantification of cell proliferation

The number of rounded cells were counted using the object detector in ImageJ. Rounded cells were detected by searching for large objects with high circularity in one dataset to determine the ideal paramaters semi-quantitatively. Parameters of minimum diameter 12 µm and 0.85 circularity were then applied to all data sets. The process of cell division (splitting) takes around 25 min, given that we used intervals of 20 minutes this should ensure accurate tracking.

### Statistical methods

We use two-tail distribution T tests with unequal variance.

## Electronic supplementary material

V1: Early stage somite

V2: Mid stage somite

V3: Late stage somite

V4: Timelapse slice, mid stage somite, 80µm deep

V5: Timelapse slice, mid stage somite, 100µm deep

V6: Early stage tracked somite

V7: Mid stage tracked somite

V8: Late stage tracked somite

Figure legends for manuscript videos
