# Supplementary material for: 4D imaging reveals stage dependent random and directed cell motion during somite morphogenesis
Source: Sci Rep. 2018 Aug 23;8:12644. doi: 10.1038/s41598-018-31014-3 (PMC6107556; doi:10.1038/s41598-018-31014-3)
Supplement: Supplementary file 9 — Figure legends for manuscript videos [file 41598_2018_31014_MOESM9_ESM.pdf]

**Supplementary information**

# 4D imaging reveals stage dependent random and directed cell motion during somite morphogenesis

James McColl[1,2,4,*], Gi Fay Mok[1,4,], Anna H Lippert[2], Aleks Ponjavic[2], Leila Muresan[3] and Andrea Münsterberg[1,*]

This document contains figure legends for the manuscript videos

V1: Early stage somite. Movie is de-noised and move from dorsal to ventral through the somite. Scale bar 20μm.

V2: Mid stage somite. Movie is de-noised and move from dorsal to ventral through the somite. Scale bar 20μm.

V3: Late stage somite. Movies are de-noised and move from dorsal to ventral through the somite. Scale bar 20μm.

V4: Timelapse slice, mid stage somite, 80μm deep. Movie is de-noised and runs over 180 minutes. The neural tube is on the left of the image. Scale bar 20μm.

V5: Timelapse slice, mid stage somite, 100μm deep. Movie is de-noised and runs over 180 minutes. The neural tube is on the left of the image. Scale bar 20μm.

V6: Early stage tracked somite. Movie is de-noised, inverted and individual cells tracked. Track move from yellow (early) through to red (late). Scale bar 20μm.

V7: Mid stage tracked somite. Movie is de-noised, inverted and individual cells tracked. Track move from yellow (early) through to red (late). Scale bar 20μm.

V8: Late stage tracked somite. Movie is de-noised, inverted and individual cells tracked. Track move from yellow (early) through to red (late). Scale bar 20μm.
